# Supplementary material for: Medical device surveillance with electronic health records
Source: NPJ Digit Med. 2019 Sep 25;2:94. doi: 10.1038/s41746-019-0168-z (PMC6761113; doi:10.1038/s41746-019-0168-z)
Supplement: Supplementary file 1 — Supplemental Material [file 41746_2019_168_MOESM1_ESM.pdf]

Supplementary Material

Supplementary Methods

Dictionary-based preprocessing

For hip implant systems, we built a dictionary of system names by querying the FDA Global Unique Device Identifier Database[1], which captures >900 hip implant components including femoral stems, femoral heads, acetabular components, and liners. For pain, we built a dictionary of 31 terms (e.g. 'pain', 'tender',) through manual inspection of notes. A complications dictionary of 452 terms was built via manual inspection of notes by clinical experts. Dictionaries were automatically expanded using an open source corpus processor[2] to capture synonyms and misspellings. The dictionary of anatomical entities consisted of all strings in the Foundational Model of Anatomy (FMA)[3], a small dictionary of informal abbreviations ("abd" -> "abdomen"), and regular expressions for standard anatomical terms of position (e.g., "*lateral* left knee").

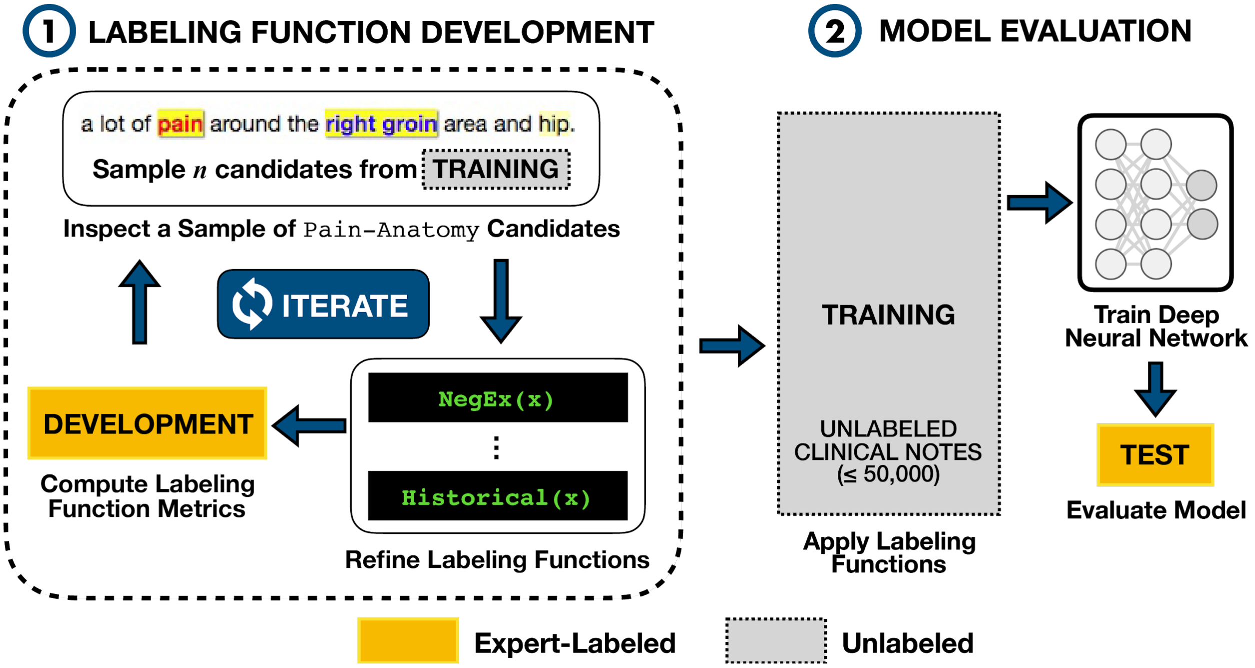

Supplementary Figure 1. The labeling function development and model evaluation workflow. In (1) domain experts examine unlabeled candidate relationships to gain insight into writing and refining labeling functions. These functions are then empirically evaluated for accuracy, precision, recall, and F1 score on an expert-labeled development set. This is an iterative process until the desired labeling function performance is achieved on the development set. In (2) the final labeling functions are applied to a large collection of unlabeled data to generate probabilistic labels for training a deep learning model. The resulting trained model is evaluated on expert-labeled unseen test set. This approach requires orders of magnitude less hand-labeled data than what would be needed for directly training deep learning model in (2), because hand-labeled data is only used to develop labeling functions and to evaluate final model performance.

Concept extraction models

By restricting our implant candidate extraction to the specific operative notes for each patient's THA procedure, we sufficiently disambiguated implant mentions to achieve high performance using dictionary-based string matching. Thus our implant candidates were used directly as our final implant outputs.

For pain extraction, we learned a generative model from labeling functions applied to unlabeled patient notes to create a probabilistically labeled training set. We then used this data to train a state-of-the-art *Bidirectional Long Short-Term Memory* (LSTM)[4] neural network with attention as our end discriminative model. Hyperparameter tuning was done using random search over 10 models, using a parameter grid derived from the literature (batch_size: {32, 128, 256}, dropout: {0.0, 0.25, 0.5}, emb_dim: {100, 300, 500}, output_layer_size: {50, 100, 400}, lstm_layers: {1,2,4}, learning_rate: [1e-4, 1e-2]).

For the final predicted pain events, all anatomical entities were normalized to UMLS concept unique identifiers (CUIs) using rule-based linking to the FMA. CUIs were linked to the most specific (i.e., longest distance to root node) concept in the FMA.

Supplementary Results

Modeling pain outcomes as relations enables detection of long-distance mentions

In our gold set, 52.51% of all Pain-Anatomy mentions occurred 1 or more words apart. At a note-level, 39% of positive pain relations occurred *only* as long distance mentions. These long distance relations also contain different information compared to compound mentions (e.g., "hip pain"): for notes containing both mention types, the anatomical locations mentioned overlap by only 15% on average.

Structured revision record-free survival among implant systems

Supplementary Figure 2 summarizes the risk of revision for implant systems when including evidence from structured records of revision only. Based on this data, no implant system is associated with a significantly higher or lower risk of revision. Supplementary Figures 3-7 summarize the risk of component wear, mechanical failure, particle disease, radiographic abnormality and infection (the complication subclasses detected by our extraction pipeline) for implant systems.

Implant System	N	Events	Person-Years	p-value	
Zimmer Biomet Trilogy + VerSys	722	27	3321.1	Reference	
Depuy Duraloc + AML	74	5	713.6	0.75 (0.27, 2.07)	0.6
Depuy Duraloc + Corail	197	3	501.8	0.74 (0.22, 2.50)	0.6
Depuy Pinnacle + AML	101	8	873.0	1.03 (0.45, 2.33)	0.9
Depuy Pinnacle + Corail	247	1	439.1	0.30 (0.04, 2.21)	0.2
Depuy Pinnacle + Endurance	38	3	320.2	1.21 (0.34, 4.26)	0.8
Depuy Pinnacle + Summit	614	12	1618.7	1.04 (0.51, 2.09)	0.9
Zimmer Biomet Continuum + M/L Taper	98	3	149.9	2.21 (0.65, 7.55)	0.2
Zimmer Biomet M2a + Osteocap	10	1	77.5	1.37 (0.18, 10.46)	0.8
Zimmer Biomet Ranawat/Burstein + Taperloc	21	1	64.7	1.99 (0.27, 14.93)	0.5
Zimmer Biomet Trilogy + Epoch	39	2	159.2	1.62 (0.38, 6.95)	0.5
Zimmer Biomet Trilogy + M/L Taper	280	5	707.6	0.89 (0.34, 2.36)	0.8
Zimmer Biomet Trilogy + Reach	16	1	74.3	1.76 (0.24, 13.19)	0.6
Zimmer Biomet Trilogy + ZMR	10	1	27.2	4.39 (0.58, 33.05)	0.2

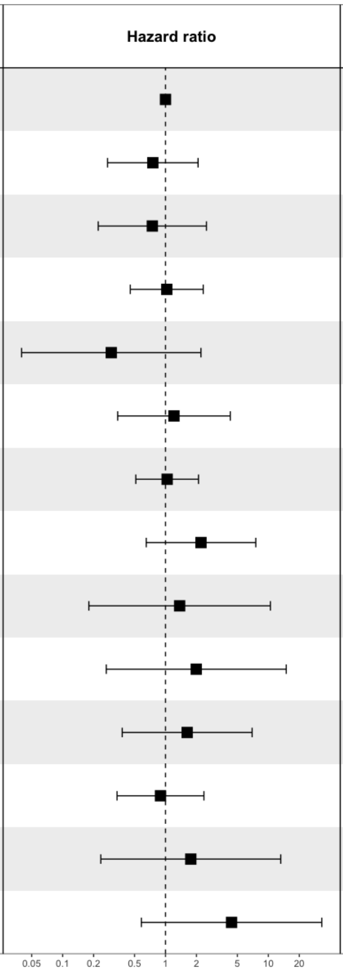

Supplementary Figure 2. Summary of Cox proportional hazards analysis of the risk of revision for each hip implant system, when including only structured records of revision. The table on the left lists the number of patients implanted with each system, the number of revision events observed for each based on structured records only, and the total person-years of data available. The forest plot displays the corresponding hazard ratio, with the hazard ratio (95% confidence interval) and p-value listed in the table to the right. Note that this figure only shows implant systems for which at least one revision event was detected.

Post-implant complication-free survival among implant systems

Supplementary Figures 3-7 summarize the risk of each class of post-implant complication for different implant systems, as derived by a Cox proportional hazards analysis.

Implant System	N	Events	Person-Years		p-value
Zimmer Biomet Trilogy + VerSys	719	46	3244.5	Reference	
Depuy Duraloc + AML	74	11	697.1	1.15 (0.57, 2.32)	0.700
Depuy Duraloc + Corail	196	10	478.2	1.07 (0.54, 2.15)	0.844
Depuy Duraloc + Summit	10	1	33.1	1.98 (0.27, 14.47)	0.503
Depuy Pinnacle + AML	101	11	826.9	0.98 (0.50, 1.93)	0.957
Depuy Pinnacle + Corail	246	6	442.1	0.68 (0.29, 1.61)	0.380
Depuy Pinnacle + Endurance	38	5	305.4	1.45 (0.55, 3.78)	0.452
Depuy Pinnacle + Summit	612	37	1560.2	1.46 (0.93, 2.29)	0.096
Depuy Pinnacle + Tri-lock	21	1	92.8	0.84 (0.12, 6.13)	0.865
Depuy Pinnacle + Zimmer Biomet Mallory-Head	11	1	85.4	0.91 (0.12, 6.64)	0.922
Stryker Trident + Zimmer Biomet Wagner	28	5	59.1	3.60 (1.40, 9.30)	0.008 *
Zimmer Biomet Continuum + Depuy Summit	2	1	0.4	18.41 (2.42, 139.74)	0.005 *
Zimmer Biomet Continuum + M/L Taper	98	7	145.7	1.67 (0.75, 3.76)	0.211
Zimmer Biomet M2a + Osteocap	10	2	77.5	2.07 (0.49, 8.73)	0.322
Zimmer Biomet Ranawat/Burstein + Taperloc	21	2	63.7	1.72 (0.41, 7.14)	0.458
Zimmer Biomet Trilogy + Advocate	37	1	124.0	0.56 (0.08, 4.11)	0.568
Zimmer Biomet Trilogy + Depuy AML	18	2	176.6	0.86 (0.21, 3.60)	0.838
Zimmer Biomet Trilogy + Epoch	39	3	150.4	1.28 (0.40, 4.15)	0.677
Zimmer Biomet Trilogy + M/L Taper	279	10	687.4	0.75 (0.38, 1.51)	0.424
Zimmer Biomet Trilogy + Reach	16	2	70.6	2.12 (0.51, 8.78)	0.301

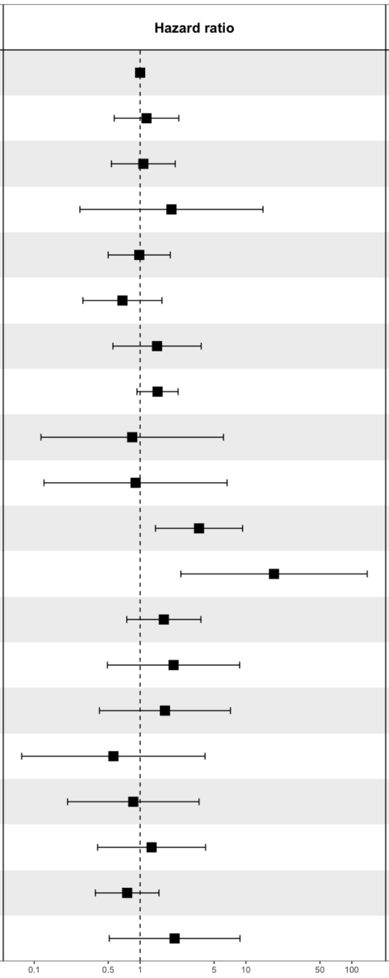

Supplementary Figure 3. Summary of Cox proportional hazards analysis of the risk of component wear for each hip implant system. The table on the left lists the number of patients implanted with each system, the number of component wear events observed for each, and the total person-years of data available. The forest plot displays the corresponding hazard ratio, with the hazard ratio (95% confidence interval) and p-value listed in the table to the right. Note that this figure only shows implant systems for which at least one component wear event was detected.

Implant System	N	Events	Person-Years	Hazard ratio		p-value
Zimmer Biomet Trilogy + VerSys	722	101	3068.0		Reference	
Depuy Duraloc + AML	74	14	660.5		0.70 (0.39, 1.26)	0.232
Depuy Duraloc + Corail	197	20	477.6		1.06 (0.65, 1.73)	0.804
Depuy Duraloc + Summit	10	1	35.7		0.86 (0.12, 6.20)	0.882
Depuy Pinnacle + AML	101	11	834.6		0.45 (0.24, 0.85)	0.013 *
Depuy Pinnacle + Corail	247	13	421.3		0.72 (0.40, 1.29)	0.273
Depuy Pinnacle + Endurance	38	5	318.0		0.48 (0.19, 1.21)	0.119
Depuy Pinnacle + Solution	6	1	32.8		1.11 (0.15, 7.99)	0.919
Depuy Pinnacle + Summit	614	27	1591.5		0.48 (0.31, 0.75)	0.001 *
Depuy Pinnacle + Tri-lock	21	1	90.6		0.34 (0.05, 2.41)	0.277
Stryker Trident + Zimmer Biomet Wagner	29	6	68.8		2.01 (0.87, 4.66)	0.103
Zimmer Biomet Continuum + Advocate	3	1	5.4		4.55 (0.63, 32.83)	0.133
Zimmer Biomet Continuum + Epoch	8	2	9.2		4.15 (1.01, 17.00)	0.048 *
Zimmer Biomet Continuum + M/L Taper	98	9	142.6		1.15 (0.58, 2.30)	0.688
Zimmer Biomet Continuum + VerSys	13	1	24.8		0.94 (0.13, 6.76)	0.948
Zimmer Biomet Converge + Zweymuller	4	1	19.1		1.64 (0.22, 12.05)	0.626
Zimmer Biomet Ranawat/Burstein + Taperloc	21	2	63.1		0.79 (0.19, 3.20)	0.737
Zimmer Biomet RingLoc + Osteocap	2	1	5.0		7.81 (1.08, 56.73)	0.042 *
Zimmer Biomet RingLoc + Taperloc	16	3	32.1		2.12 (0.67, 6.73)	0.204
Zimmer Biomet Trilogy + Advocate	37	5	117.8		1.13 (0.45, 2.82)	0.796
Zimmer Biomet Trilogy + Depuy AML	18	1	179.7		0.19 (0.03, 1.38)	0.100
Zimmer Biomet Trilogy + Epoch	39	6	153.0		1.12 (0.49, 2.56)	0.793
Zimmer Biomet Trilogy + M/L Taper	280	17	672.2		0.61 (0.36, 1.02)	0.062
Zimmer Biomet Trilogy + Reach	16	1	74.3		0.44 (0.06, 3.16)	0.414
Zimmer Biomet Trilogy + ZMR	10	1	27.3		0.83 (0.12, 5.99)	0.855

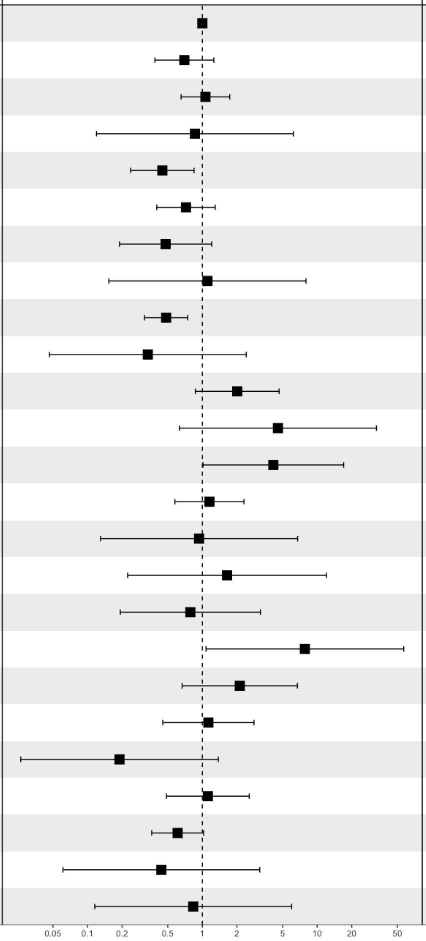

Supplementary Figure 4. Summary of Cox proportional hazards analysis of the risk of mechanical failure for each hip implant system. The table on the left lists the number of patients implanted with each system, the number of mechanical failure events observed for each, and the total person-years of data available. The forest plot displays the corresponding hazard ratio, with the hazard ratio (95% confidence interval) and p-value listed in the table to the right. Note that this figure only shows implant systems for which at least one mechanical failure event was detected.

Implant System	N	Events	Person-Years	Hazard ratio		p-value
Depuy Pinnacle + Summit	525	1	1376.3		Reference	
Depuy Duraloc + Corail	174	1	429.6		5.20 (0.21, 126.09)	0.31
Depuy Pinnacle + Tri-lock	15	1	64.9		30.32 (1.62, 567.67)	0.02 *
Zimmer Biomet Continuum + M/L Taper	74	1	111.8		29.87 (0.81, 1103.48)	0.07
Zimmer Biomet Ranawat/Burstein + Taperloc	17	1	49.3		89.97 (2.52, 3208.20)	0.01 *

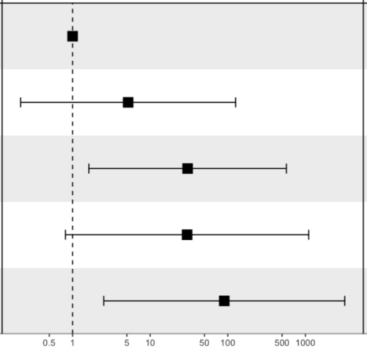

Supplementary Figure 5. Summary of Cox proportional hazards analysis of the risk of particle disease for each hip implant system. The table on the left lists the number of patients implanted with each system, the number of particle disease events observed for each, and the total person-years of data available. The forest plot displays the corresponding hazard ratio, with the hazard ratio (95% confidence interval) and p-value listed in the table to the right. Note that this figure only shows implant systems for which at least one particle disease event was detected.

Implant System	N	Events	Person-Years	Hazard ratio		p-value
Zimmer Biomet Trilogy + VerSys	724	135	2941.8		Reference	
Depuy Duraloc + AML	74	12	671.7		0.40 (0.21, 0.74)	0.003 *
Depuy Duraloc + Corail	198	31	441.5		1.25 (0.84, 1.86)	0.274
Depuy Duraloc + Solution	3	1	18.7		0.86 (0.12, 6.35)	0.883
Depuy Duraloc + Summit	10	1	37.4		0.56 (0.08, 4.05)	0.569
Depuy Pinnacle + AML	101	12	847.4		0.33 (0.18, 0.61)	<0.001 *
Depuy Pinnacle + Corail	247	27	412.6		1.11 (0.73, 1.69)	0.637
Depuy Pinnacle + Endurance	38	8	298.8		0.61 (0.29, 1.27)	0.182
Depuy Pinnacle + Summit	614	35	1582.6		0.46 (0.32, 0.67)	<0.001 *
Depuy Pinnacle + Tri-lock	21	4	80.7		1.10 (0.40, 2.97)	0.858
Stryker Trident + Zimmer Biomet Wagner	29	7	64.0		1.82 (0.84, 3.94)	0.130
Zimmer Biomet Continuum + Epoch	8	3	9.4		3.79 (1.19, 12.07)	0.024 *
Zimmer Biomet Continuum + M/L Taper	98	15	132.8		1.47 (0.86, 2.53)	0.162
Zimmer Biomet Continuum + VerSys	13	2	25.1		1.26 (0.31, 5.11)	0.749
Zimmer Biomet Continuum + Wagner	7	1	7.1		1.64 (0.23, 11.84)	0.625
Zimmer Biomet Converge + Zweymuller	4	1	19.1		1.14 (0.16, 8.27)	0.899
Zimmer Biomet M2a + Osteocap	10	4	62.2		1.60 (0.58, 4.38)	0.361
Zimmer Biomet Ranawat/Burstein + Taperloc	21	3	59.0		0.90 (0.29, 2.85)	0.861
Zimmer Biomet RingLoc + Taperloc	16	3	34.9		1.62 (0.51, 5.10)	0.414
Zimmer Biomet Trilogy + Advocate	37	7	110.4		1.29 (0.60, 2.80)	0.515
Zimmer Biomet Trilogy + Depuy AML	18	6	140.6		1.02 (0.45, 2.34)	0.954
Zimmer Biomet Trilogy + Epoch	39	7	138.5		1.05 (0.49, 2.26)	0.897
Zimmer Biomet Trilogy + M/L Taper	280	42	620.4		1.19 (0.84, 1.70)	0.333
Zimmer Biomet Trilogy + Reach	16	3	74.6		0.92 (0.29, 2.90)	0.887
Zimmer Biomet Trilogy + ZMR	10	3	23.5		2.13 (0.67, 6.74)	0.200

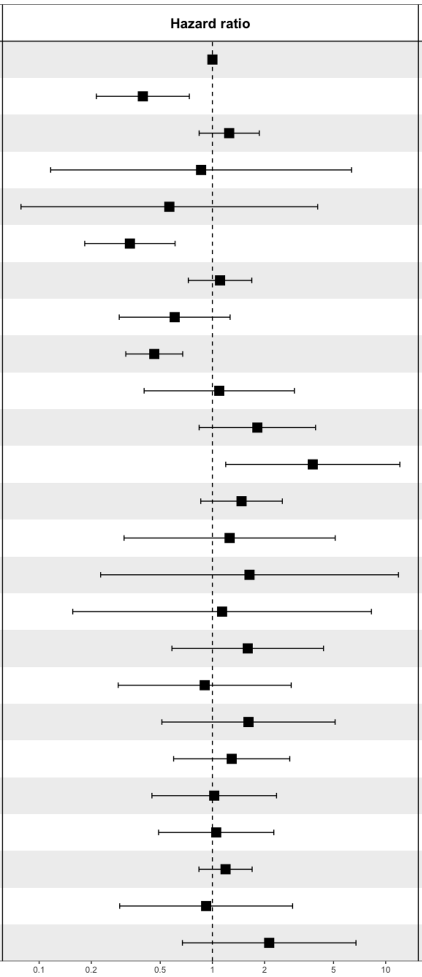

Supplementary Figure 6. Summary of Cox proportional hazards analysis of the risk of radiographic abnormality for each hip implant system. The table on the left lists the number of patients implanted with each system, the number of radiographic abnormality events observed for each, and the total person-years of data available. The forest plot displays the corresponding hazard ratio, with the hazard ratio (95% confidence interval) and p-value listed in the table to the right. Note that this figure only shows implant systems for which at least one radiographic abnormality event was detected.

Implant System	N	Events	Person-Years		p-value
Zimmer Biomet Trilogy + VerSys	724	43	3283.2	Reference	
Depuy Duraloc + AML	74	1	742.1	0.10 (0.01, 0.77)	0.027 *
Depuy Duraloc + Corail	198	17	473.3	2.39 (1.34, 4.28)	0.003 *
Depuy Pinnacle + AML	101	6	877.2	0.60 (0.25, 1.43)	0.247
Depuy Pinnacle + Corail	247	6	436.6	0.85 (0.36, 2.03)	0.716
Depuy Pinnacle + Endurance	38	3	326.4	0.90 (0.27, 3.04)	0.866
Depuy Pinnacle + Summit	614	15	1628.0	0.70 (0.38, 1.28)	0.249
Depuy Pinnacle + Tri-lock	21	1	90.5	0.86 (0.12, 6.31)	0.884
Stryker Trident + Zimmer Biomet Wagner	29	2	73.6	1.86 (0.44, 7.82)	0.397
Zimmer Biomet Continuum + M/L Taper	98	3	146.7	0.95 (0.29, 3.10)	0.928
Zimmer Biomet Continuum + Wagner	7	1	7.3	6.11 (0.81, 46.27)	0.080
Zimmer Biomet Converge + Zweymuller	4	1	18.5	4.23 (0.57, 31.16)	0.157
Zimmer Biomet M2a + Osteocap	10	2	69.4	2.16 (0.51, 9.11)	0.293
Zimmer Biomet Ranawat/Burstein + Taperloc	21	1	64.4	0.98 (0.13, 7.17)	0.984
Zimmer Biomet RingLoc + Osteocap	2	1	6.8	9.67 (1.17, 79.85)	0.035 *
Zimmer Biomet Trilogy + Advocate	37	2	122.9	1.09 (0.26, 4.62)	0.903
Zimmer Biomet Trilogy + Depuy AML	18	2	160.5	0.91 (0.21, 3.96)	0.900
Zimmer Biomet Trilogy + Epoch	39	1	160.8	0.47 (0.06, 3.42)	0.454
Zimmer Biomet Trilogy + M/L Taper	280	13	676.6	1.18 (0.62, 2.22)	0.613
Zimmer Biomet Trilogy + Reach	16	2	77.8	2.04 (0.49, 8.54)	0.328
Zimmer Biomet Trilogy + ZMR	10	1	31.1	1.89 (0.26, 13.96)	0.531

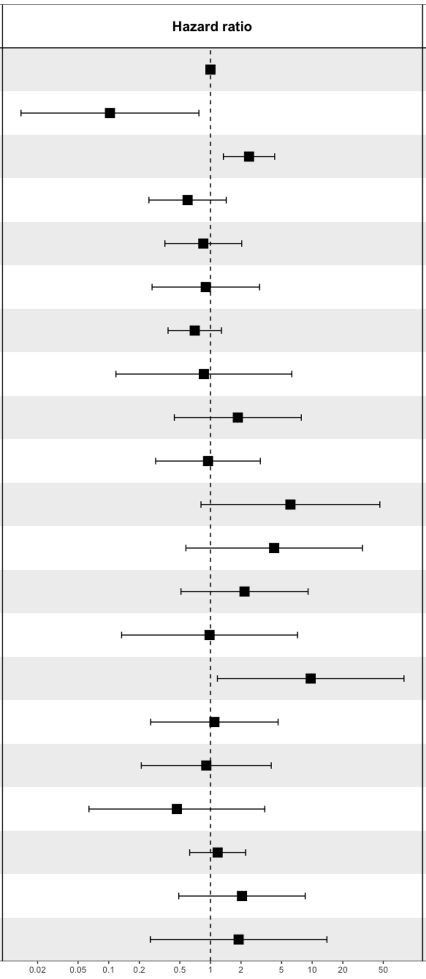

Supplementary Figure 7. Summary of Cox proportional hazards analysis of the risk of infection for each hip implant system. The table on the left lists the number of patients implanted with each system, the number of infection events observed for each, and the total person-years of data available. The forest plot displays the corresponding hazard ratio, with the hazard ratio (95% confidence interval) and p-value listed in the table to the right. Note that this figure only shows implant systems for which at least one infection event was detected.

Post-implant hip pain is associated with implant system

Supplementary Table 1 lists the model coefficient estimate, incident rate ratio (IRR), lower and upper 95% confidence interval bounds, and p-values for the negative binomial model of hip pain in the year post-THA.

Supplementary Table 1. Negative binomial model coefficients, IRR (95% confidence interval) and p-value for hip pain in the year after THA.

Variable	Estimate	IRR (95% CI)	p-value
(Intercept)	0.828	2.290 (1.455-3.604)	< 0.001 *
**Implant System**			
Zimmer Biomet Trilogy + VerSys	Reference		
Depuy Duraloc + AML	-3.415	0.033 (0.012-0.091)	< 0.001 *
Depuy Duraloc + Corail	0.238	1.268 (1.020 -1.577)	0.033 *
Depuy Duraloc + Summit	-1.827	0.161 (0.040-0.652)	0.011 *
Depuy M2A + Osteocap	0.408	1.504 (0.640-3.537)	0.350
Depuy Pinnacle + AML	-2.334	0.097 (0.057-0.164)	< 0.001 *
Depuy Pinnacle + Corail	-0.521	0.594 (0.475-0.742)	< 0.001 *
Depuy Pinnacle + Endurance	-3.431	0.032 (0.008-0.137)	< 0.001 *
Depuy Pinnacle + Solution	-0.417	0.659 (0.199-2.187)	0.496
Depuy Pinnacle + Summit	-1.020	0.361 (0.301-0.432)	< 0.001 *
Depuy Pinnacle + TriLock	-0.524	0.592 (0.300-1.171)	0.132
Zimmer Biomet Continuum + Epoch	0.530	1.700 (0.679-4.257)	0.258
Zimmer Biomet Continuum + M/L Taper	0.723	2.061 (1.561-2.720)	< 0.001 *
Zimmer Biomet Continuum + VerSys	0.589	1.802 (0.881-3.685)	0.107
Zimmer Biomet Continuum + Wagner	-0.352	0.703 (0.242-2.047)	0.518
Zimmer Biomet Trilogy + Depuy AML	-0.411	0.663 (0.321-1.367)	0.266
Zimmer Biomet Trilogy + Epoch	0.313	1.368 (0.884-2.117)	0.160
Zimmer Biomet Trilogy + M/L Taper	0.399	1.490 (1.234-1.799)	< 0.001 *
Zimmer Biomet Trilogy + Reach	-0.345	0.708 (0.335-1.499)	0.367
Zimmer Biomet Trilogy + Wagner	0.607	1.834 (1.120-3.004)	0.016 *
Zimmer Biomet Trilogy + ZMR	-0.237	0.789 (0.319-1.953)	0.608
Other system	-0.278	0.757 (0.504-1.138)	0.181
**Charlson Comorbidity Index**			
None	Reference		

Low	0.141	1.151 (0.956-1.386)	0.137
Moderate	0.040	1.040 (0.809-1.338)	0.758
High	0.234	1.264 (0.997-1.601)	0.053
**Age**			
40-49 years	Reference		
50-59 years	-0.035	0.965 (0.794-1.174)	0.723
60-69 years	0.050	1.051 (0.870-1.269)	0.606
70-79 years	-0.062	0.940 (0.768-1.151)	0.549
80+ years	-0.219	0.803 (0.632-1.020)	0.072
**Sex**			
Female	Reference		
Male	0.010	1.010 (0.898-1.137)	0.862
**Race**			
Asian	Reference		
Black	0.108	1.114 (0.726-1.709)	0.620
Native American	-0.171	0.843 (0.171-4.162)	0.834
Other	0.394	1.482 (1.047-2.099)	0.027 *
Pacific Islander	0.357	1.430 (0.598-3.420)	0.422
Unknown	0.053	1.055 (0.684-1.626)	0.810
White	-0.030	0.970 (0.751-1.253)	0.817
**Ethnicity**			
Hispanic	Reference		
Not Hispanic	0.036	1.036 (0.746-1.439)	0.832
Unknown	-0.719	0.487 (0.328-0.723)	< 0.001 *
**Other covariates**			
Pain in year prior to THA	0.071	1.073 (1.049-1.099)	< 0.001 *
Follow-up time	-0.001	0.999 (0.999-1.000)	0.001 *

Relation extraction system performance

Supplementary Table 2 details Pain-Anatomy extraction performance given 150 - 50,000 weakly labeled training documents. Here we see performance improvements up to +9.2 F1 points over soft majority vote as we increase the scale of weakly labeled data provided to the deep learning model.

Supplementary Table 2. `Pain-Anatomy` Relation Extraction Performance

Model	Training Set Size (Number of Documents)											
	150			5K			10K			50K		
	P	R	F1	P	R	F1	P	R	F1	P	R	F1
Supervised-LSTM ✦	72.5	78.4	75.4	-	-	-	-	-	-	-	-	-
Soft Majority Vote	**81.4**	64.8	72.2	-	-	-	-	-	-	-	-	-
Weakly Supervised LSTM	68.4	81.8	74.5	75.1	80.5	77.7	76.4	80.9	78.6	80.2	**82.6**	**81.4**

✦ Uses hand-labeled training data
Blue highlighting Highest achieved value for metric (P, R, F1)

Supplementary Table 3 contains a non-exhaustive list of example terms for each Implant-Complication category. These terms form disjoint sets for each Implant-Complication sub-category.

Supplementary Table 3. Example Terms for `Implant-Complication` Subcategories

Subcategory	Terms
Mechanical failure	hardware loosening, crooked, asymmetrically seated
Revision	reoperation, removals, revision, rebuilt, hardware removal
Component wear	polyethylene wear, worn, wearing, bearing surface wear, debonding
Infection	infection, septic, abscess, re-infected
Particle disease	particle disease, metal ion toxicity, metallosis
Radiographic abnormality	lucencies, pedestals, heterotopic calcifications, spurs

Supplementary Table 4 contains full performance metrics for all extracted relations, including Implant-Complication sub-categories. Confidence intervals are computed using test set bootstrapping with n=1000 replicates.

Supplementary Table 4. Performance Metrics for all Relations

Complication Type	Mentions	BASELINE Soft Majority Vote			Fully Supervised (n= 150 docs)			Weakly Supervised Model			+/- F1
	N	P (SD)	R (SD)	F1 (SD)	P (SD)	R (SD)	F1 (SD)	P (SD)	R (SD)	F1 (SD)	%
**Pain-Anatomy**	236	**81.4 (2.8)**	64.8 (3.0)	72.1 (2.3)	72.5 (2.9)	78.3 (2.6)	75.3 (2.1)	80.2 (2.6)	**82.5 (2.4)**	**81.3 (1.9)**	+12.8
**Implant-Complication**	276	81.6 (3.6)	31.7 (2.7)	45.6 (3.1)	50.8 (3.1)	47.1 (3.1)	48.8 (2.7)	**82.6 (2.6)**	**61.1 (2.9)**	**70.2 (2.3)**	+53.9
Revision	63	74.4 (6.9)	45.8 (5.9)	56.5 (5.7)	41.8 (4.8)	68.2 (6.0)	51.7 (4.7)	**75.6 (6.1)**	**58.6 (6.0)**	**65.9 (5.1)**	+16.6
Component Wear	48	71.1 (8.8)	42.4 (7.3)	52.8 (7.1)	**78.4 (9.8)**	31.7 (7.0)	44.8 (7.9)	72.7 (6.5)	**72.9 (6.4)**	**72.9 (5.3)**	+38.1
Mechanical Failure	25	87.1 (13.2)	27.8 (9.5)	41.3 (11.4)	21.5 (7.6)	27.3 (9.2)	23.7 (7.7)	**90.9 (8.8)**	**43.6 (10.2)**	**58.2 (10.1)**	+40.9
Particle Disease	65	80.2 (19.5)	6.2 (3.0)	11.6 (5.2)	54.1 (7.9)	32.5 (5.9)	40.3 (6.1)	**97.1 (2.8)**	**52.5 (6.0)**	**68.0 (5.2)**	+486.2
Radiographic Abnormality	17	**99.8 (4.5)**	**33.5 (10.9)**	**49.1 (12.4)**	37.2 (9.2)	55.9 (12.2)	44.1 (9.3)	59.7 (16.6)	35.4 (12.0)	43.6 (12.5)	-12.6
Infection	58	**100.0 (0.0)**	39.8 (6.5)	56.6 (6.7)	90.1 (4.6)	62.0 (6.5)	73.3 (5.2)	90.8 (4.0)	**84.6 (4.9)**	**87.5 (3.4)**	+54.6

References

1. U S Food And Drug Administration. Global UDI Database (GUDID). Available at: https://www.fda.gov/MedicalDevices/DeviceRegulationandGuidance/UniqueDeviceIdentification/GlobalUDIDatabaseGUDID/default.htm. (Accessed: 30th March 2018)
2. Paumier, S., Nakamura, T. & Voyatzi, S. UNITEX, a Corpus Processing System with Multi-Lingual Linguistic Resources. eLEX2009 (2009).
3. Rosse, C. & Mejino, J. L. V., Jr. A reference ontology for biomedical informatics: the Foundational Model of Anatomy. J. Biomed. Inform. **36**, 478–500 (2003).
4. Zhou, P. et al. Attention-based bidirectional long short-term memory networks for relation classification. in Proceedings of the 54th Annual Meeting of the Association for Computational Linguistics (Volume 2: Short Papers) **2**, 207–212 (2016).
